# Conditional Inhibition of Eip75B Eliminates the Effects of Mating and Mifepristone on Lifespan in Female *Drosophila*

**DOI:** 10.3390/cells13131123

**Published:** 2024-06-28

**Authors:** Gary N. Landis, Hans S. Bell, Oscar K. Peng, Yijie Fan, Karissa Yan, Britta Baybutt, John Tower

**Affiliations:** Molecular and Computational Biology Section, Department of Biological Sciences, University of Southern California, Los Angeles, CA 90089-2910, USA

**Keywords:** aging, *Drosophila*, mifepristone, plasticity, steroid, PPARγ

## Abstract

Mating in female *Drosophila melanogaster* causes midgut hypertrophy and reduced lifespan, and these effects are blocked by the drug mifepristone. Eip75B is a transcription factor previously reported to have pleiotropic effects on *Drosophila* lifespan. Because Eip75B null mutations are lethal, conditional systems and/or partial knock-down are needed to study Eip75B effects in adults. Previous studies showed that Eip75B is required for adult midgut cell proliferation in response to mating. To test the possible role of Eip75B in mediating the lifespan effects of mating and mifepristone, a tripartite FLP-recombinase-based conditional system was employed that provides controls for genetic background. Expression of a *Hsp70-FLP* transgene was induced in third instar larvae by a brief heat pulse. The FLP recombinase catalyzed the recombination and activation of an *Actin5C-GAL4* transgene. The GAL4 transcription factor in turn activated expression of a *UAS-Eip75B-RNAi* transgene. Inhibition of Eip75B activity was confirmed by loss of midgut hypertrophy upon mating, and the lifespan effects of both mating and mifepristone were eliminated. In addition, the negative effects of mifepristone on egg production were eliminated. The data indicate that Eip75B mediates the effects of mating and mifepristone on female midgut hypertrophy, egg production, and lifespan.

## 1. Introduction

The synthetic steroid drug mifepristone (RU486) has a history of safe and effective use in humans [1,2,3]. Mifepristone is used as a treatment for Cushing’s syndrome (hypercortisolism) based upon its antagonism of the type II glucocorticoid receptor (GR). Mifepristone is also extensively used for birth control based upon its antagonism of the progesterone receptor. Recent studies report that mifepristone also exhibits anti-diabetic and anti-obesity effects in both mice and humans [4,5,6,7,8,9,10]. For example, short-term treatment of obese human patients with mifepristone reduced serum triglyceride levels and improved tissue insulin sensitivity [6]. Mifepristone has been found to be an agonist of mammalian PPARγ [11,12], and therefore, both PPARγ and the GR are implicated as relevant targets for the beneficial effects of mifepristone on mammalian metabolism. Strikingly, mifepristone produces similar effects in the model organism *Drosophila*, including decreased triglyceride levels and inhibition of female reproduction [13].

During the process of mating in *Drosophila*, the male introduces a seminal hormone called sex peptide (SP) into the female reproductive tract [14]. In the female, the SP causes increased levels of the steroid hormone ecdysone and a hormone called juvenile hormone (JH). The ecdysone and JH in turn cause dramatic changes in the females’ physiology, including midgut hypertrophy, increased lipid and amino acid metabolism, increased triglyceride levels, increased egg production, inflammation, and decreased lifespan [15,16,17,18,19,20,21,22,23]. Notably, all of these effects are largely or completely reversed by treating the mated females with mifepristone, resulting in dramatic increases in median lifespan [22,24,25]. Mifepristone also increases lifespan and reduces triglyceride levels and amino acid metabolite levels in virgin females but does not detectably affect virgin female midgut size [13,26]. In addition, mifepristone does not increase lifespan in males [24].

The effect of mifepristone on food intake has been assayed using several methods. The dye-uptake assay [27] was used to assay food intake in mated females of three different genotypes [24]. Treatment with mifepristone caused no decrease in food intake in any genotype and significantly increased food intake in one genotype. The CAFÉ assay [28] was used to assay food intake in mated females of two genotypes [22]. Treatment with mifepristone caused no decrease in food intake in either genotype and significantly increased food intake in one genotype. The excrement quantification (EXQ) assay [29] was used to assay food intake in virgin females of one genotype in three different studies [25,26,30]. Treatment with mifepristone did not cause a decrease in food intake in any assay and significantly increased food intake in one assay. Taken together, the data indicate that mifepristone does not reduce food intake in either virgin females or mated females, regardless of genotype, and instead is often associated with increased food intake. The increased food intake might be a result of improved health of the mifepristone treated flies, and/or might represent a compensatory response to the reduced lipid and amino acid metabolism caused by mifepristone. In contrast, mifepristone is reported to have an aversive effect under extremely low nutrient conditions, which may be relevant to studies of dietary restriction [31]. Mifepristone increases lifespan of virgin and mated females under both normal and axenic conditions and has no detectable antibiotic effects [32,33]. Mifepristone was first used in *Drosophila* as the trigger for the conditional gene expression systems called Gene-Switch and P[Switch], based upon its ability to bind and activate the engineered Gene-Switch transcription factor [34,35,36,37]. It is important to note that the effects of mifepristone on lifespan, reproduction, gene expression, and metabolism are observed in the absence of the Gene-Switch transcription factor [22,24,25,26].

Mifepristone produces smaller but still significant increases in lifespan in female flies where the germline and egg laying have been ablated by a maternal *tudor[1]* mutation [13]. This indicates that reduced egg production might contribute to the lifespan benefits of mifepristone, but other mechanisms must also be involved, such as the reductions in intestine size, metabolism, and inflammation. Several results suggest that midgut lipid uptake and lipid metabolism may be particularly important. For example, the *Drosophila* Dhr96 hormone receptor is orthologous to the human vitamin D receptor and LXR (NR1H) receptor [38,39]. Dhr96 functions in the Drosophila midgut to promote lipid uptake and lipid metabolism, and *Dhr96[1]* null mutant animals have reduced triglyceride levels [40,41,42]. The *Dhr96[1]* mutation was found to increase lifespan, and to reduce but not eliminate the lifespan effects of mating and mifepristone [13]. These results suggest that *Dhr96[1]* mutation and mifepristone may increase lifespan in part through the same mechanism of reducing lipid metabolism. Consistent with this model, lipidomics analysis revealed that mifepristone reduces whole-body levels of triglycerides and fatty acids in both virgin and mated females [13]. The human drug etomoxir inhibits the rate-limiting enzyme for the transport of long-chain FAs into the mitochondria, carnitine palmitoyltransferase I (CPT I) [43]. Similar to mifepristone, etomoxir increased lifespan in virgin females and mated females, but not in males [30]. Taken together, these data support the hypothesis that the mechanism for mifepristone lifespan increase may involve reduced midgut lipid uptake and/or metabolism.

As mentioned above, mifepristone binds and activates the mammalian PPARγ hormone receptor. *Drosophila* contains an ortholog of PPARγ called Eip75B [38]. Eip75B is increasingly implicated in the regulation of lifespan and mating responses in *Drosophila* and is therefore a strong candidate for a possible *Drosophila* mifepristone receptor. *Eip75B* null mutations are lethal during development [44] and therefore cannot be assayed for adult phenotypes. However, a P element insertion mutation in *Eip75B* was viable, and was associated with small increases in lifespan in both male and mated female flies [45]. Similarly, combining a *UAS-Eip75B-RNAi* construct with a weak, tissue-general GAL4 driver produced small increases in lifespan in both male and mated female flies [46]. In addition, naturally occurring sequence variants of *Eip75B* have been associated with increased lifespan in males and mated females [46,47]. In contrast, combining a *UAS-Eip75B RNAi* construct with a GAL4 driver specific to the ovary and accessory glands produced small decreases in lifespan in mated female flies [48]. Finally, Hoedjes et al. used the Gene-Switch system to drive expression of several *UAS-Eip75B-RNAi* constructs specifically in adult flies, using the tissue-general *da*-Gene-Switch driver [49]. They observed decreases in lifespan and egg laying in mated female flies upon activation of transgene expression with mifepristone, as well as negative effects of mifepristone itself on egg laying [49]. Taken together, the studies indicate a role for Eip75B in regulation of lifespan in males and mated females. However, the direction of the lifespan effect in mated females remains unclear, and studies employing the Gene-Switch system are complicated by the effects of mifepristone, including the negative effect of mifepristone on reproduction.

Previous studies show that mating causes increased ovarian production of ecdysone, which in turn acts through the ecdysone receptor to cause increased expression of Eip75B in the midgut [15,16,19]. The requirement for Eip75B function in midgut cell proliferation and midgut hypertrophy was tested using *UAS-Eip75B-RNAi* constructs, midgut-specific GAL4 drivers, and the GAL4/GAL80ts system. In the GAL4/GAL80ts system [50,51], GAL80ts represses GAL4 activity until the temperature is shifted to 29 °C, whereupon GAL80ts becomes inactive, and GAL4 now activates target transgene expression. These studies showed a requirement for normal Eip75B function for midgut cell proliferation and normal egg laying, however, the possible effect on lifespan of Eip75B RNAi expression in intestinal cells was not assayed. Our recent studies show that the 29 °C temperature required for the GAL4/GAL80ts system significantly reduces the effects of mating and mifepristone on midgut hypertrophy, and virtually eliminates the effects of mating and mifepristone on lifespan [30], making the GAL4/GAL80ts system non-optimal for exploring the effects of Eip75B on these phenotypes. Here, an alternative conditional system called FLP-out [52,53,54] is used to overcome these limitations, and the experiments reveal a role for Eip75B in mediating the effects of mating and mifepristone on female *Drosophila* lifespan, midgut hypertrophy and egg production.

## 2. Materials and Methods

### 2.1. Drosophila Strains, Culture, Drug Treatments, and Lifespan Assay

*Drosophila melanogaster* flies were cultured at 25 °C using a standard agar/dextrose/corn meal/yeast media [13,55]. Several *Drosophila* strains were provided by the Bloomington *Drosophila* Stock Center. These include strain y[1] w[*] P{w[+mC]=GAL4-Act5C(FRT.CD2).P}D (BDSC#4779) and strain P{ry[+t7.2]=hsFLP}12, y[1] w[*]; *sna[Sco]/CyO* (BDSC#1929), abbreviated here as *yw*;*HS-FLP*;*Sco/CyO.* The *w[1118]* reference strain (*w[1118]-iso*; *2-iso*; *3-iso*) is as previously described [56]. The y[1] w[*] P{w[+mC]=GAL4-Act5C(FRT.CD2).P}D strain was backcrossed 11 generations to the *w[1118]* strain and is named as *w[1118]* *FLP-out-GAL4*. One *Drosophila* strain was obtained from the Vienna *Drosophila* Resource Center, *w[1118]*; *P{GD1434}v44851* (VDRC#v44851) and is referred to here as *UAS-75B-RNAi*. For lifespan assays, the female progeny were collected as virgins over 24 h. To generate mated females, the virgins were placed in vials with young (1–2 weeks of age) *w[1118]* strain males at a ratio of 20 males to 20 females for 48 h. The males were then removed, and all flies were maintained in culture vials in the presence/absence of drug, with every-other-day passage to fresh vials, as indicated. Mifepristone (RU486) was obtained from Sigma-Aldrich (St. Louis, MO, USA, cat. #M8046). Mifepristone treatments were conducted as described [13]; 50 μL of 20× stock solution (4 mg/mL) of mifepristone in ethanol was applied evenly to the surface of drug treatment vials, and an equal volume of ethanol vehicle was applied to control vials, and the solutions were allowed to absorb and dry for 48 h. Final concentration of mifepristone in the media was calculated as 200 μg/mL based on dye-absorption controls as previously described [55,57]. Flies were passaged to fresh +/− drug vials every other day. The number of dead flies was counted at each passage, and median lifespan, percent change in median, log-rank tests and COX proportional hazards analyses (COX-PHA) were conducted using the R statistical environment [58]. The log-rank analyses were corrected for any multiple comparisons using a Bonferroni correction. The *p* value for significance at 5% error rate is presented in the figure legends.

### 2.2. FLP-Out Activation of E75B-RNAi

The strain *yw HS-FLP12*; *UAS-75B-RNAi/CyO* was generated by crossing *yw HS-FLP12*; *Sco/CyO* virgin females to *w[1118]/Y*; *UAS-75B-RNAi* males, to yield *yw HS-FLP12/Y*; *UAS-75B-RNAi/CyO* male progeny. These males were then crossed to *yw HS-FLP12*; *Sco/CyO* virgin females, and the male and virgin female progeny bearing *CyO* and lacking *Sco* were crossed inter se to generate the final strain. To generate the experimental group females for lifespan assay, virgin females of the *yw HS-FLP12*; *UAS-75B-RNAi/CyO* strain were crossed to *w[1118]* *FLP-out-GAL4* males to generate *yw HS-FLP12/w[1118]* *FLP-out-GAL4*; *UAS-75B-RNAi/+* female progeny. To generate control group females lacking the *FLP-out-GAL4* transgene, virgin females of the *yw HS-FLP12*; *UAS-75B-RNAi/CyO* strain were crossed to *w[1118]* males to generate *yw HS-FLP12/w[1118]*; *UAS-75B-RNAi/+* female progeny. To conduct FLP-out and transgene activation from third instar larval stage onwards, both types of crosses were conducted using 20 female parents and 20 male parents per vial. The flies were allowed to lay eggs in vials for 24 h and then removed. The vials were then maintained at 25 °C. At 5 days post-egg-laying, all vials were placed in a 37 °C water bath for 1 h. The cotton stopper was pushed halfway into the vial such that any wandering larvae were kept below the water line. The heat pulse was then repeated 24 h later. Virgin female progeny were collected from the vials, and lifespan assays were conducted as described above. Because the *FLP-out-GAL4* transgene was previously backcrossed to the *w[1118]* strain for 11 generations, the experimental group and the control group have the same genetic background and differ only in the presence or absence of *FLP-out-GAL4.* Mating and drug treatment were conducted as described above. In separate experiments, the FLP-out and transgene activation were activated by heat pulses of young adult females. The same crosses were conducted as described above, and virgin females were collected over 24 h. All flies were subjected to 90-min heat pulse on Day 2 and on Day 3 using 37 °C water baths. Prior to each heat pulse, the flies were transferred to empty vials containing a folded ½ Kimwipe wetted with 0.5 mL 1% sucrose solution to avoid any flies sticking to the media during the heat pulse, and the vial stopper was pushed halfway into the vial such that all flies were below the water line. Mating, drug treatments, and lifespan assays were then conducted as described above.

### 2.3. FLP-Out without E75B-RNAi Target Transgene

Experiments were conducted to test for possible lifespan effects of FLP-out recombination in the absence of the *UAS-75B-RNAi* target transgene. To generate the experimental group females for lifespan assay, virgin females of the *yw*; *HS-FLP*; *Sco/CyO* strain were crossed to *w[1118]* *FLP-out-GAL4* males to generate *yw HS-FLP12/w[1118]* *FLP-out-GAL4*; *Sco/+* female progeny. To generate control group females lacking the *FLP-out-GAL4* transgene, virgin females of the *yw*; *HS-FLP*; *Sco/CyO* strain were crossed to *w[1118]* males to generate *yw HS-FLP12/w[1118]*; *Sco/+* female progeny. Both types of cross were conducted using 20 female parents and 20 male parents per vial. The flies were allowed to lay eggs in the vials for 24 h and were then removed. The vials were then maintained at 25 °C. At 5 days post-egg-laying, all vials were placed in a 37 °C water bath for 1 h. This was repeated 24 h later. Virgin female progeny were then collected from each vial, and lifespan assays were conducted as described above. Because the *FLP-out-GAL4* transgene was previously backcrossed to *w[1118]* strain for 11 generations, the experimental group and the control group have the same genetic background and differ only in the presence or absence of *FLP-out-GAL4.* Mating and drug treatment were conducted as described above.

### 2.4. Midgut Diameter Assay

Maximum midgut diameter was assayed in virgin female and mated female flies, at Day 12 +/− drug treatment, as previously described [26]. Briefly, midgut tissue from 5 flies at a time was dissected in PBS, and the tissue samples were then transferred to a glass slide with the coverslip spaced using double-stick tape [59]. Images were generated using visible light. The maximum midgut diameter was measured using Image J. The statistical test was unpaired, two-sided *t* test, and any outliers were identified using the Grubbs test in Prism 9. A Bonferroni correction was used to control for multiple comparisons, and the *p* value for significance at 5% error rate is indicated in the figure legends

### 2.5. Egg Laying Assay

To quantify egg production, 5 vials of 20 flies each per group were assayed at each time point, and the dissecting microscope was used to count total eggs laid per vial over 24 h, with assays beginning at ~11 AM and ending at the same time on the next day. The data are presented as average eggs per fly per day, and area under curve (AUC) analysis was used to estimate total eggs laid over the whole assay period. AUC was calculated for each of 5 replicate vials. The average standard deviation of the AUC is presented in bar graphs. Unpaired, two-sided *t* tests were used to determine any significant differences in AUC between samples using Prism 9. A Bonferroni correction was used to control for multiple comparisons, and the *p* value for significance at 5% error rate is indicated in the figure legends.

### 2.6. Weight Assay

For each group, four replicates of 10 flies each were weighed in pre-weighed microcentrifuge tubes. The average and standard deviation were calculated for each group, and the groups were compared using unpaired, two-sided *t* tests. A Bonferroni correction was used to control for multiple comparisons, and the *p* value for significance at 5% error rate is indicated in the figure legend.

### 2.7. Quantitative Real-Time PCR Assay

Gene expression was analyzed in whole-body RNA isolated from mated female flies at 14 days of age. RNA was extracted from 6 samples of 20 flies each for each group, using the PureLink RNA mini kit from Invitrogen (Cat#121183018A) and eluted with 100 μL RNAse-free water. The quantitative real-time PCR analysis was conducted by Azenta Life Sciences (South Plainfield, NJ, USA). Three technical replicates were assayed for each of the 6 biological replicates for a total of 18 assays per group. Gene expression levels were calculated using the 2^−ΔΔCT^ method [60]. The TBP gene was used as the house-keeping control, using TBP primers as previously described [49]. The *Eip75B* primers are as previously described [61]. The GAL4 primers are as previously described [62]. All primer efficiencies were confirmed using melting curve analyses. Data are presented in bar graphs as the average and standard deviation of the 18 assay values. The statistical test is an unpaired two-sided *t* test. The analysis was corrected for multiple comparisons using Bonferroni correction, and the *p* value for significance at 5% error rate is indicated in the figure legends.

## 3. Results

### 3.1. Confounding Effects of 37 °C Temperature Pulse in Young Adult Flies

Experiments were designed to test the potential role of Eip75B in mediating the effects of mating and mifepristone on adult female fly lifespan. Because null mutations of the Eip75B gene are lethal during development, a conditional system was needed to reduce Eip75B function specifically in adults. Previously, the GAL4/GAL80ts system was tested, wherein transgene expression is induced by a shift from 25 °C to 29 °C. However, the 29 °C temperature was found to be sufficient to nearly eliminate the lifespan effects of mating and mifepristone, even in the absence of any transgene induction, making that approach not useful for the desired experiments [30]. As an alternative, the FLP-out approach was tested. The FLP-out system used here involves three transgenes. In the first transgene (*Hsp70-FLP*), the heat-inducible Hsp70 promoter drives expression of the yeast FLP recombinase. In the second transgene, the tissue-general *Actin5C* promoter drives expression of yeast GAL4 transcription factor (*Actin5C-FLP-out-GAL4*). However, a transcriptional stop sequence is placed between the *Actin5C* promoter and the GAL4 ORF, so that GAL4 is not expressed. The stop sequence is flanked by FLP recombination target sequences (FRTs), such that if recombination is induced by FLP, the stop sequence is excised, and the GAL4 protein is then constitutively expressed from that point in time onwards. Finally, the third transgene is the *UAS-Eip75B-RNAi* construct, that is activated by GAL4. In this way, a brief heat pulse of 37 °C will cause expression of FLP, FLP-out of the stop sequence, and constitutive expression of *UAS-Eip75B-RNAi* from that point in time onwards.

The FLP-out system was first tested in young adult female flies. Virgin females were subjected to 37 °C heat pulse on Days 2 and 3 of age. Mating was then conducted for 48 h, the males were removed, and lifespan was assayed. The results were compared to a control group that did not receive the heat pulse. In the no-heat pulse controls, mating significantly decreased lifespan, and this was rescued by treatment with mifepristone, as expected (Figure S1; Table S1). Unfortunately, the heat pulse was found to eliminate the lifespan effects of mating and mifepristone, even in the absence of target transgene induction (Figure S1; Table S1). These results indicate that, similar to a constant 29 °C temperature, even transient pulses of 37 °C temperature in young adult females is sufficient to inhibit the lifespan effects of mating and mifepristone, meaning that this system is not useful for the desired experiments when used with young adult flies.

### 3.2. FLP-Out Transgene Activation in Third Instar Larvae

**Figure 1:**
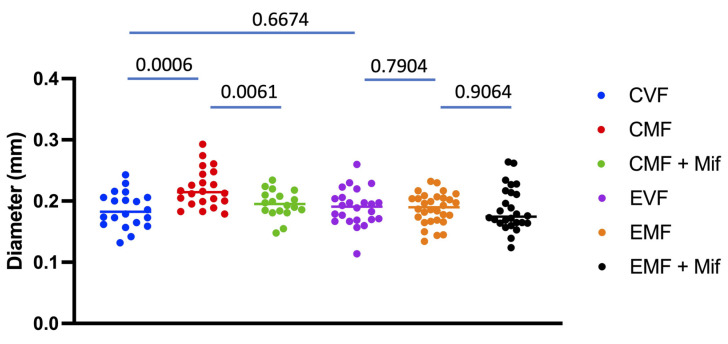
Conditional transgene activation eliminates mating-induced midgut hypertrophy. All flies were subjected to heat pulses at the third-instar larvae stage. Virgin females (VF), mated females (MF), and mated females treated with mifepristone (MF + Mif) were then assayed for maximum midgut diameter at an age of 14 days. Control group females (CVF, CMF) contain the control chromosome. Genotype *yw HS-FLP12/w[1118]*; *UAS-75B-RNAi/+*. Experimental group females (EVF, EMF) contain the isogenic chromosome with FLP-out target transgene. Genotype *yw HS-FLP12/w[1118]* *FLP-out-GAL4*; *UAS-75B-RNAi/+*. Statistical test is unpaired two-sided *t*-test, and the Bonferroni-corrected *p* value for significance with two comparisons is *p* < 0.025.

To avoid the confounding effects of heat pulse observed in young adult flies, experiments were conducted by subjecting third instar larvae to the heat pulse. Experimental group flies containing all three constructs were compared to control group flies that lacked the *Actin5C-FLP-out-GAL4* construct. Because the *Actin5C-FLP-out-GAL4* strain has previously been backcrossed to the *w [1118]* reference strain for 11 generations, the experimental and control group flies had the same genetic background (Section 2). Expression of this specific *UAS-Eip75B-RNAi* construct has previously been shown to inhibit midgut cell proliferation in young adult female flies [15,16]. Both the experimental and control groups were subjected to 37 °C heat pulses for 1 h on Days 5 and 6 post-egg-laying to target third-instar larvae. The resultant adult flies were then assayed for phenotypic effects. Effective inhibition of Eip75B activity was confirmed by assaying midgut hypertrophy at an age of 14 days in virgin female flies and mated female flies. In the control group flies, mating caused robust midgut hypertrophy, which was reduced by mifepristone treatment, as expected, whereas in the experimental group flies, the mating-induced midgut hypertrophy and the effects of mifepristone were eliminated (Figure 1). Examples of midgut tissue are presented for the control group (Figure S2), and for the experimental group (Figure S3). A 2-way ANOVA of virgin females and mated females confirmed a significant effect of mating, a significant effect of genotype, and a significant mating-by-genotype interaction (Table S2). In addition, a 2-way ANOVA of (−) Mif and (+) Mif females confirmed a significant effect of Mif, a significant effect of genotype, and a significant Mif-by-genotype interaction (Table S3). Therefore, the data indicate that by conducting the heat pulse in third-instar larvae, the confounding effects of the heat pulse are avoided and Eip75B function is effectively reduced.

**Figure 2:**
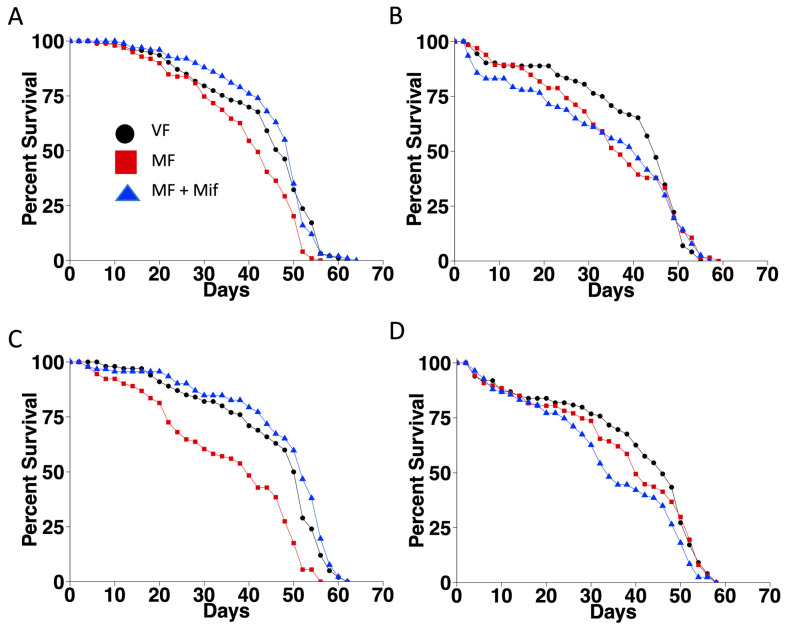
Conditional transgene activation eliminates the effects of mating and mifepristone on lifespan. All flies were subjected to heat pulses at the third larval instar stage. Virgin females (VF), mated females (MF), and mated females treated with mifepristone (MF + Mif) were assayed for lifespan in two replicate experiments. (**A**,**C**) Control group females containing control chromosome. Genotype *yw HS-FLP12/w[1118]*; *UAS-75B-RNAi/+*. (**B**,**D**) Experimental group females containing isogenic chromosome with FLP-out target transgene. Genotype *yw HS-FLP12/w[1118]* *FLP-out-GAL4*; *UAS-75B-RNAi/+*. (**A**,**B**) Experiment 1. (**C**,**D**) Experiment 2. Statistical summary presented in Table 1.

Next, lifespan was assayed in two replicate assays of the control group and two replicate assays of the experimental group. In the control group, mating significantly reduced lifespan (−12.5% and −21.6%, respectively), and mifepristone rescued lifespan (+19% and +30%, respectively), as expected (Figure 2A,C; Table 1). Notably, in the experimental group, no significant life span effect of mating or mifepristone was detected (Figure 2B,D; Table 1). It is also noteworthy that inhibition of Eip75B was associated with an overall reduction in life span. For example, life span of virgin females was reduced by −6.25% in Experiment 1 and by −9.8% in Experiment 2 (compare EVF to CVF; Table 1).

The log-rank test allows only one variable to be analyzed at a time for its effect on survival. In contrast, the Cox proportional hazards regression analysis (COX-PHA) allows multiple variables to be analyzed simultaneously for their effects on survival, and reveals whether or not these variables have significant interactions [63]. The data from Experiment 1 and Experiment 2 were combined and analyzed by COX-PHA (Table 2). The regression coefficient (coef) indicates how each variable is weighted with regard to risk of death. The z score (Wald statistic) is the ratio of the coefficient to its standard error (z = coef/se(coef)). This statistic reveals whether the coefficient is significantly different than zero. A positive coefficient and z score indicates that the variable increases risk of death, whereas a negative coefficient and z score indicate the variable reduces the risk of death. Consistent with the results of the log-rank tests, the COX-PHA reveals that mating increases the risk of death, whereas mifepristone reduces the risk of death. The COX-PHA also shows the significant effect of genotype and the inhibition of Eip75B on survival. The presence of the *Flp-out-GAL4* transgene (FOG4) was associated with increased risk of death, consistent with the observation made above that the inhibition of Eip75B reduces overall lifespan. In addition, there was a significant interaction between FOG4 and mating that reduced the risk of death, consistent with the conclusion that inhibition of Eip75B eliminates the negative effect of mating on lifespan. Finally, there was a significant interaction between FOG4 and mifepristone that increased the risk of death, consistent with the conclusion that inhibition of Eip75B eliminates the positive effect of mifepristone on lifespan. Taken together, these experiments and analyses indicate that normal Eip75B function is required to observe the effects of mating and mifepristone on lifespan.

### 3.3. FLP Recombination Does Not Significantly Alter Lifespan

**Figure 3:**
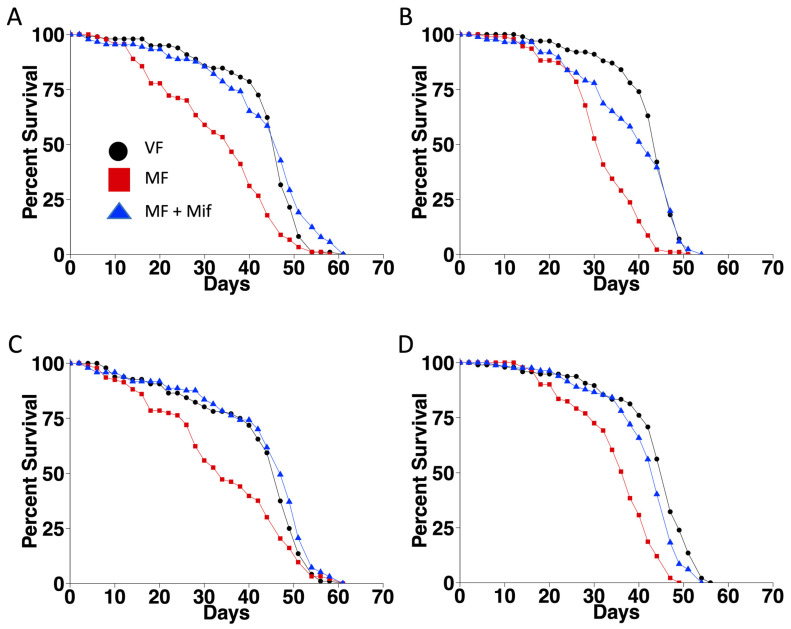
Effect of FLP-out recombination on lifespan in the absence of a target gene. All flies were subjected to heat pulses at the third larval instar stage. Virgin females (VF), mated females (MF), and mated females treated with mifepristone (MF + Mif) were assayed for lifespan in two replicate experiments. (**A**,**C**) Control group females containing control chromosome. Genotype *yw HS-FLP12/w[1118]; Sco/+*. (**B**,**D**) Experimental group females containing isogenic chromosome with FLP-out target transgene. Genotype *yw HS-FLP12/w[1118]* *FLP-out-GAL4; Sco/+*. (**A**,**B**) Experiment 1. (**C**,**D**) Experiment 2. Statistical summary presented in Table 3.

In the experiments presented above, all flies were subjected to the same heat pulse treatments, and the control and experimental groups differed in the absence and presence of the *Actin5C-FLP-out-GAL4* construct, respectively. To confirm that the process of FLP recombination itself does not inhibit the lifespan effects of mating and mifepristone, additional experiments were conducted on control and experimental groups that differed in the absence and presence of the *Actin5C-FLP-out-GAL4* construct, in the absence of the *UAS-Eip75B-RNAi* target construct. Fly culture and heat pulses were conducted as described above, and lifespan was assayed in virgin females, mated females, and mated females treated with mifepristone in two replicate experiments (Figure 3; Table 3). In the control group, containing only *Hsp70-FLP*, mating significantly reduced lifespan (−23.4% and −27.7%, respectively), and mifepristone rescued lifespan (+30.1% and +38.2%, respectively), as expected. In the experimental group, containing *Hsp70-FLP* and *Actin5C-FLP-out-GAL4*, similar results were obtained. Mating significantly decreased lifespan (−27.3% and −19.1%, respectively), and mifepristone rescued lifespan (+31.3% and +15.8%, respectively). Comparing the lifespans of the control and experimental groups using COX-PHA confirmed the significant negative effect of mating and the significant positive effect of mifepristone and confirmed no significant effect of FLP-out (FOG4) over the whole dataset (Table 3). The COX-PHA also showed no significant interaction between FLP-out and mifepristone, consistent with the conclusion that FLP-out does not reduce the effects of mifepristone on lifespan. The COX-PHA did show a significant interaction between FLP-out and mating that reduced the risk of death (Table 4). However, there was no consistent direction of effect of FLP-out on lifespan in the mated females detected using log-rank tests (Table 3). Taken together, these experiments indicate that the process of FLP recombination itself does not significantly alter the lifespan effects of mifepristone. However, it may have a small and variable effect in reducing the negative effects of mating.

### 3.4. Effect of FLP-Out Transgene Activation on Egg Production

**Figure 4:**
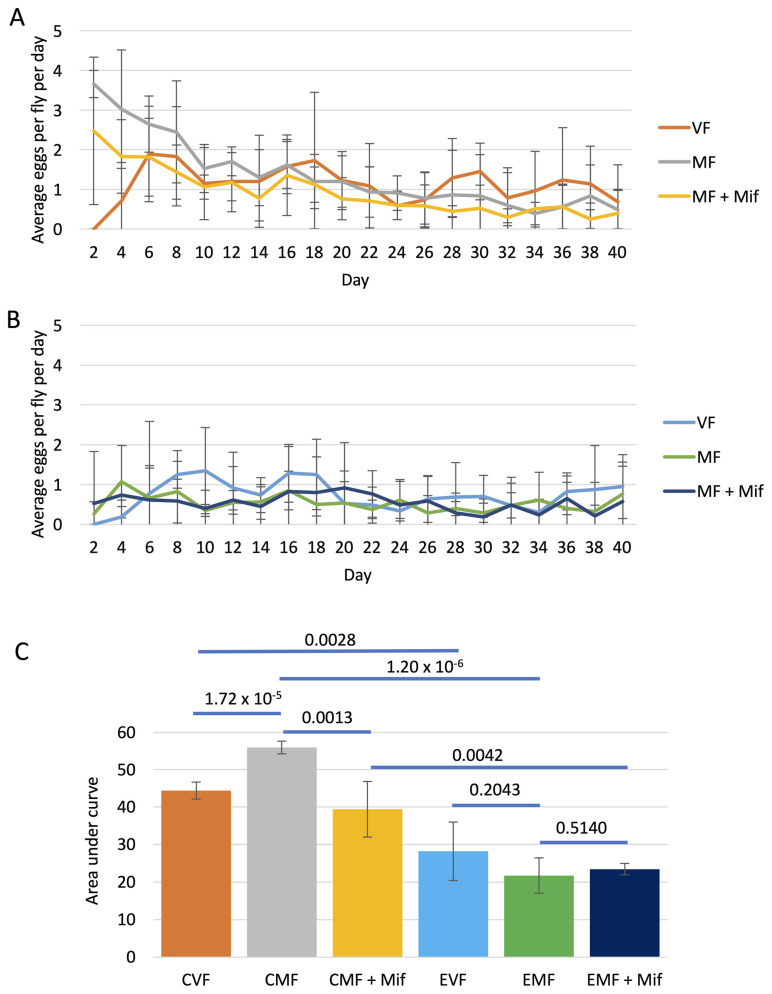
Effect of conditional transgene activation on egg production. All flies were subjected to heat pulses at third larval instar stage. Virgin females (VF), mated females (MF), and mated females treated with mifepristone (MF + Mif) were then assayed for egg production at regular time points, as indicated. Error bars indicate average and standard deviation of 5 replicate vials per group. (**A**) Control group females containing control chromosome. Genotype *yw HS-FLP12/w[1118]*; *UAS-75B-RNAi/+*. (**B**) Experimental group females containing isogenic chromosome with FLP-out target transgene. Genotype *yw HS-FLP12/w[1118]* *FLP-out-GAL4*; *UAS-75B-RNAi/+*. (**C**) Area under curve (AUC) analysis. The AUC was calculated for each replicate vial, and the average and standard deviation for each group is presented in the bar graph. CVF, control group virgin females. CMF, control group mated females. EVF, experimental group virgin females. EMF, experimental group mated females. Statistical test is unpaired two-sided *t*-test, and the Bonferroni-corrected *p* value for significance with three comparisons is *p* < 0.0167.

To ask if FLP-out activation of Eip75B RNAi might affect egg production and the effect of mifepristone, egg production was assayed in virgin females, mated females, and mated females treated with mifepristone. Egg production was assayed at regular time points from Day 2 to Day 40, in control group flies containing only the *Hsp70-FLP* and *UAS-Eip75B-RNAi* constructs (Figure 4A), as well as in experimental group flies containing all three constructs (*Hsp70-FLP*, *Actin5C-FLP-out-GAL4* and *UAS-Eip75B-RNAi*; Figure 4B). AUC analysis was then used to estimate the total egg production across the assay period (Figure 4C). In the control group, mifepristone significantly reduced egg production, consistent with our previous studies [13,24]. In contrast, in the experimental group, mifepristone had no significant effect on egg production, indicating that normal Eip75B function is required for mifepristone to reduce egg production. Notably, egg production was reduced in the experimental group virgin females relative to virgin control females, and egg production was also reduced in experimental group mated females relative to control group mated females, indicating that normal Eip75B activity is required for normal egg production in both virgin and mated females (Figure 4C).

**Figure 5:**
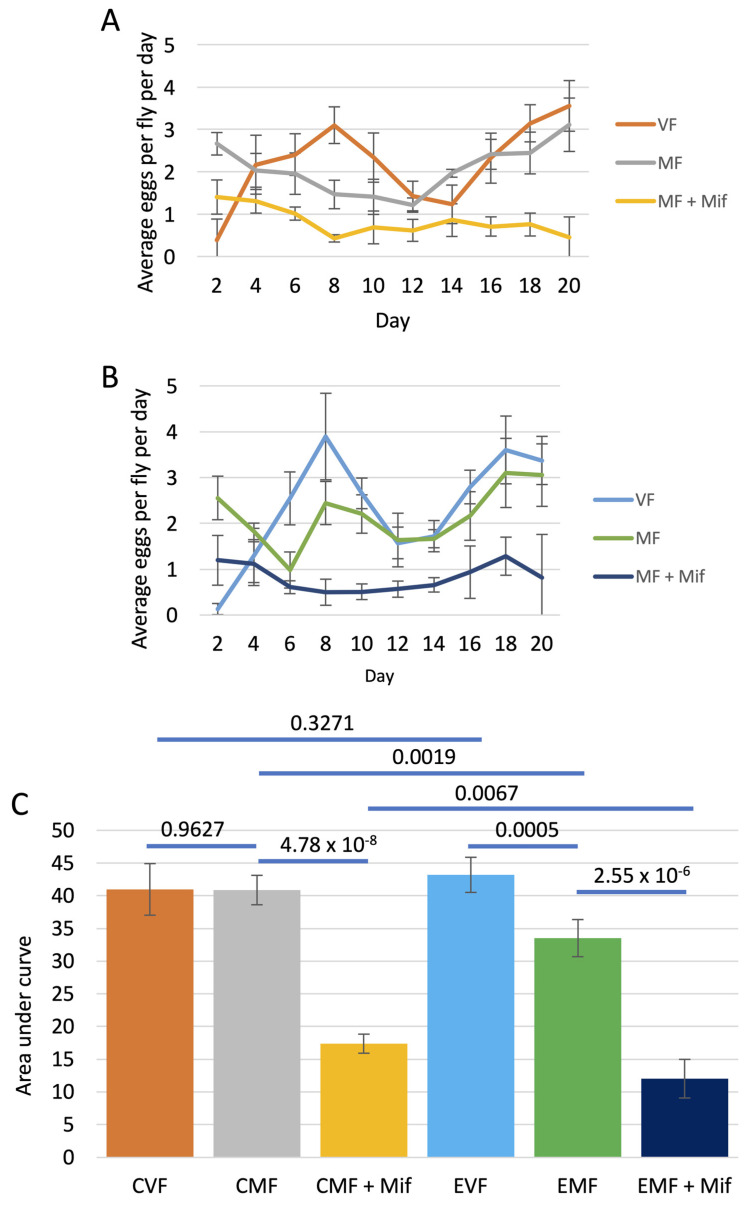
Effect of FLP-out recombination on egg production in the absence of a target gene. All flies were subjected to heat pulses at third larval instar stage. Virgin females (VF), mated females (MF), and mated females treated with mifepristone (MF + Mif) were then assayed for egg production at regular time points, as indicated. Error bars indicate average and standard deviation of 5 replicate vials per group. (**A**) Control group females containing control chromosome. Genotype *yw HS-FLP12/w* *[1118]*; *Sco/+.* (**B**) Experimental group females containing isogenic chromosome with FLP-out target transgene. Genotype *yw HS-FLP12/w[1118]* *FLP-out-GAL4*; *Sco/+.* (**C**) Area under curve (AUC) analysis. The AUC was calculated for each replicate vial, and the average and standard deviation for each group is presented in the bar graph. CVF, control group virgin females. CMF, control group mated females. EVF, experimental group virgin females. EMF, experimental group mated females. Statistical test is unpaired two-sided *t*-test, and the Bonferroni-corrected *p* value for significance with three comparisons is *p* < 0.0167.

Control experiments were also conducted to ask if the process of FLP recombination might affect egg production and response to mifepristone. Egg production was assayed in virgin females, mated females, and mated females treated with mifepristone, at regulator time points from Day 2 to Day 20. Control group females containing only the *Hsp70-FLP* construct were compared to experimental group females containing the *Hsp70-FLP* construct and the *Actin5C-FLP-out-GAL4* construct. Mifepristone significantly reduced egg production in mated females of the control group as well as in mated females of the experimental group, indicating that FLP-recombination does not significantly alter the negative effects of mifepristone on egg production (Figure 5).

### 3.5. Effect of FLP-Out Transgene Activation and FLP-Out Recombination on Fly Weight

**Figure 6:**
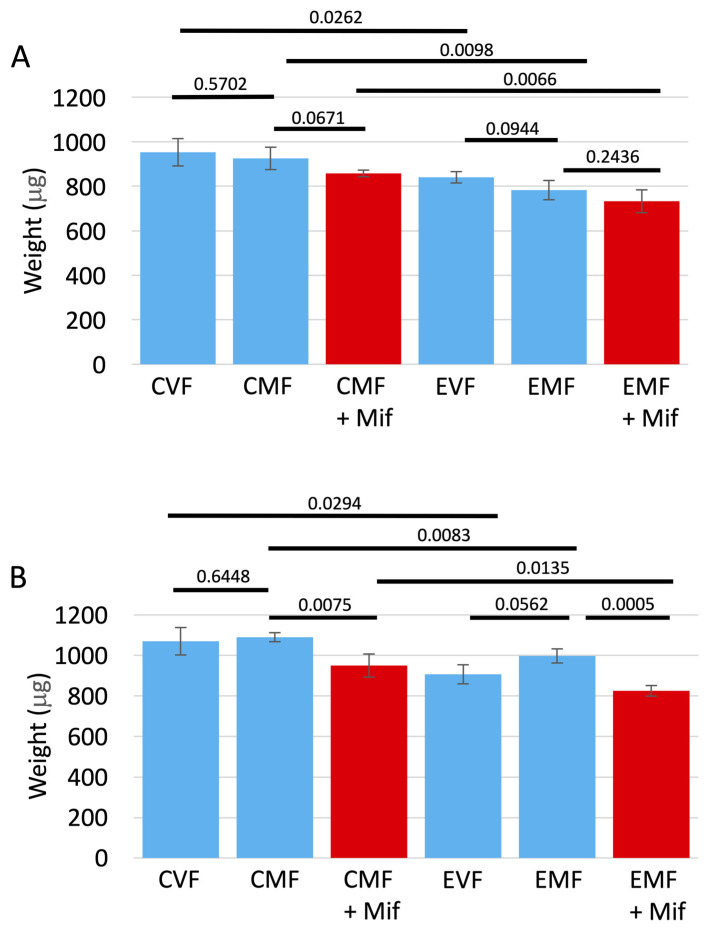
Fly weight. All flies were subjected to heat pulses at the third larval instar stage. Virgin females (VF), mated females (MF), and mated females treated with mifepristone (MF + Mif) were then assayed for weight at an age of 14 days. (**A**) Eip75B-RNAi containing genotypes. The control groups (CVF, CMF) contained the control chromosome, genotype *yw HS-FLP12/w[1118]*; *UAS-75B-RNAi/+*. The experimental groups (EVF, EMF) contained the isogenic FLP-out target transgene, genotype *yw HS-FLP12/w[1118]* *FLP-out-GAL4*; *UAS-75B-RNAi/+*. (**B**) Genotypes lacking *Eip75B-RNAi*. The control group (CVF, EMF) contained the control chromosome, genotype *yw HS-FLP12/w[1118]*; *Sco/+*, and the experimental group (EVF, EMF) contained the isogenic chromosome with FLP-out target transgene, *genotype yw HS-FLP12/w[1118]* *FLP-out-GAL4*; *Sco/+*. Error bars indicate average and standard deviation of 4 replicates of 20 flies each per group. Statistical test is unpaired two-sided *t*-test, and the Bonferroni-corrected *p* value for significance with three comparisons is *p* < 0.0167.

Experiments were conducted to determine if FLP-out activation of Eip75B RNAi might affect fly weight. Fly weight was assayed at an age of 14 days in virgin females, mated females, and mated females treated with mifepristone. To determine the possible effect of Eip75B inhibition, genotypes were assayed that contained the *UAS-Eip75B-RNAi* transgene (Figure 6A). In control group flies (CVF, CMF) that lacked the *FLP-out-GAL4* transgene, mating and mifepristone treatment had no statistically significant effect on weight. Similarly, in the experimental group flies (EVF, EMF) that contained the *Flp-out-GAL4* transgene, mating and mifepristone again had no significant effect on weight. However, EMF flies had significantly reduced weight relative to CMF flies, indicating that inhibition of Eip75B was associated with an ~17% decrease in mated female weight. To determine whether this effect was indeed due to Eip75B inhibition as opposed to an effect of the FLP-out process itself, genotypes were assayed that lacked the *UAS-Eip85B-RNAi* transgene (Figure 6B). In control group flies (CVF, CMF) that lacked the *FLP-out-GAL4* transgene, mating had no statistically significant effect on weight, however, mifepristone caused a significant decrease of ~13%. Similarly, in the experimental group flies (EVF, EMF) that contained the *Flp-out-GAL4* transgene, mating had no statistically significant effect on weight, however, mifepristone caused a significant decrease of ~19%. Comparing CMF flies to EMF flies revealed that the presence of the *Flp-out-GAL4* transgene was associated with an ~8% decrease in weight. Therefore, the ~17% decrease in mated female weight associated with Eip75B inhibition (Figure 6A), may or may not represent an effect in addition to the ~8% weight reduction in mated females associated with the FLP-out process itself (Figure 6B). The observation that mifepristone did not have a statistically significant effect on mated female weight in the genotypes that contained the *UAS-Eip75B-RNAi* transgene (Figure 6A), but mifepristone did have a significant effect on mated female weight in the genotypes that lacked the *UAS-Eip75B-RNAi* transgene (Figure 6B), might suggest that normal Eip75B function is required for mifepristone to affect weight. However, because mifepristone significantly affected mated female weight in the absence of the *Flp-out-GAL4* transgene (Figure 6B), it suggests that the different responses to mifepristone observed are due to variation in the assay and/or other minor differences in genetic background between the two types of experiments.

### 3.6. Quantitative Real-Time PCR Assay

A qPCR assay was used to quantify gene expression using whole-body RNA isolated from mated females at 14 days of age. Transcript levels were normalized to the house-keeping gene TBP. The control group contained the control chromosome, genotype *yw HS-FLP12/w[1118]*; *UAS-75B-RNAi/+*, and the experimental group contained the isogenic FLP-out target transgene, genotype *yw HS-FLP12/w[1118]* *FLP-out-GAL4*; *UAS-75B-RNAi/+*. All animals were subjected to heat pulses at the third instar larval stage. Experimental group flies exhibited a dramatic induction of GAL4 mRNA, as expected (Figure S4A). However, no significant differences in Eip75B mRNA levels were detected when comparing control and experimental groups (Figure S4B). Given the robust inhibition of Eip75B function observed above, as indicated by the expected loss of midgut hypertrophy, we conclude that high levels of Eip75B mRNA from one or more tissues that are not efficiently targeted by the RNAi may be obscuring a loss of Eip75B expression in some critical tissue(s) where the RNAi is more effective.

## 4. Discussion

Here, the FLP-out conditional system was used to activate expression of a target transgene beginning at the third larval instar stage. The resultant adult female flies were then assayed for midgut diameter, lifespan and egg production. The FLP-out system provides powerful controls for environment and genetic background. Both the control group and experimental group flies receive an identical and coincident heat pulse, thereby removing temperature effects as a possible confounding variable. The control group and experimental group flies have the same genetic background and differ only in the presence of the FLP-out transgene in the experimental group. Therefore, FLP recombination and target gene activation can only occur in the experimental group. In control flies, mating caused increased midgut diameter, increased egg production, and decreased lifespan, and these effects were largely or completely blocked by treatment with mifepristone, consistent with our previous studies [13,22,24,25,26]. FLP-out activation of the *Eip75B* RNAi transgene in the experimental group flies was found to eliminate the effects of mating and mifepristone on each phenotype. These results indicate that normal Eip75B function is required for the effects of mating and mifepristone on midgut diameter, egg production and lifespan, and support the hypothesis that Eip75B protein might be a direct target of mifepristone. Conceivably, inhibition of Eip75B could eliminate the negative effect of mating on lifespan by making mated females live longer or by reducing the lifespan of virgin females and mated females plus mifepristone to the same level as mated females. Comparing lifespan between the control and experimental groups reveals that inhibition of Eip75B reduces overall lifespan, consistent with the latter possibility.

Ahmed et al. recently showed that expression of *Eip75B-RNAi* specifically in midgut cells reduced egg production, and therefore, Eip75B does appear to be required in the midgut for normal egg production [16]. However, this does not mean that Eip75B is only required in the midgut. Indeed, previous studies have reported a requirement for Eip75B in the germ line cells for normal egg production [64,65]. The FLP-out system used here drives expression of GAL4 using the tissue-general *Actin5C* gene promoter, and therefore expression of the *UAS-Eip75B-RNAi* transgene and inhibition of Eip75B function is expected to occur in all somatic tissues. In the future, it will be important to determine the critical tissue(s) for Eip75B function in regulating the responses to mating and mifepristone.

Notably, all experimental group flies had significantly reduced egg production relative to their corresponding control group flies. Virgin female egg production was reduced by 36%, mated female egg production was reduced by 62%, and mated female treated with mifepristone egg production was reduced by 41% (Figure 4C). These results are consistent with previous studies that showed that Eip75B is required in somatic ovarian cells for normal egg production in mated females [64,66]. In addition, these results are consistent with a requirement for Eip75B in the midgut cells for mating-induced midgut hypertrophy and increased lipid production to support normal egg production [16,19].

As discussed above, in the present experiments, the control and experimental groups differ in that FLP-out recombination and target transgene activation occurs only in the experimental group. Therefore, additional controls were conducted to test for any possible effects of FLP recombination in the absence of a target transgene. Both log rank tests and COX-PHA showed that FLP recombination had little or no significant effect on lifespan. Similarly, AUC quantification of egg laying indicated that FLP recombination did not affect egg laying in virgin females. However, FLP recombination was associated with a -17% reduction in egg production in mated females (Figure 5C; compare CMF to EMF). Therefore, we cannot rule out the possibility that a small negative effect of FLP recombination on egg laying (−17%) contributes to the larger negative effect observed for FLP recombination plus target gene activation on egg laying (−62%) in the mated female group.

Whereas mating was associated with increased egg laying in the control genotype that contained the Eip75B RNAi transgene (Figure 4C), mating was not associated with increased egg laying in the control genotypes that lacked the *Eip75B-RNAi* transgene (Figure 5C). Consistent with previous studies [67], we find that when increased egg laying in mated females relative to virgin females is observed, it is driven almost entirely by increases in the first ~5 days post-mating (Figure 5A). This early egg laying has sometimes been referred to as “egg dumping” and is modulated by variables including male seminal hormones as well as intergenerational effects [67,68,69]. We conclude that this difference in results between the two experiments is due to variability in the assay and/or minor differences between the genotypes.

Interestingly, in the genotypes that contained the *Flp-out-GAL4* transgene, mating was associated with a decrease in egg production. This reduction was not statistically significant in the genotype containing the *Eip75B-RNAi* transgene (Figure 4C) but did reach statistical significance in the genotype lacking the *Eip75B-RNAi* transgene (Figure 5C). These results suggest some negative interaction between the process of FLP-out and egg production that is observed in mated females but not virgin females (Figure 5C); the possible reason for this effect is not clear at this time. The fact that this effect was reduced in the genotype where Eip75B function was inhibited may be because those flies already had greatly reduced egg production in virgin females (Figure 4C).

The requirement for normal Eip75B activity for mating-induced increases in midgut size and egg production suggests that increased midgut nutrient uptake and metabolism resulting from the increase in midgut size supports the increased egg production [16,19], and our results are consistent with this model. However, FLP-out inhibition of Eip75B activity did not alter midgut diameter in virgin females (Figure 1; compare CVF to EVF) but did significantly reduce virgin female egg laying, indicating that Eip75B plays a role in virgin egg production that is independent of changes in midgut size. One possibility is that Eip75B acts in the midgut of virgin females to promote lipid metabolism that is required for normal virgin female egg production. In the future, it may be of interest to further explore the role of Eip75B in virgin female physiology, including lifespan, and to ask if Eip75B might affect food intake.

Previous studies show that mating causes increased ovarian production of ecdysone, which in turn acts through the ecdysone receptor to cause increased expression of Eip75B in midgut progenitor cells. The increased Eip75B activity then promotes ISC proliferation and midgut hypertrophy [15,16]. Both ecdysone and the ecdysone receptor have been reported to negatively affect female *Drosophila* lifespan [70,71], and consistent with that observation, shortened lifespan caused by the powerful ecdysone mimic RH5849 is partly rescued by mifepristone [30]. However, mifepristone does not reduce the expression of an ecdysone receptor-responsive reporter in control flies, or when the reporter is stimulated by feeding the flies with ecdysone or RH5849, indicating that mifepristone does not directly inhibit the ecdysone receptor [25,30]. The fact that mifepristone can block the effects of mating on midgut hypertrophy, egg production, and lifespan and that the effects of mifepristone are eliminated by inhibition of Eip75B supports the hypothesis that Eip75B might be a mifepristone target.

Maternal *tudor[1]* mutation produces offspring that lack the germline and produce no eggs [72,73]. Previously, these sterile females were shown to have reduced but still significant changes in lifespan due to mating and mifepristone, indicating that the germline and changes in egg production are not essential for the effects of mifepristone [13]. These results are therefore generally consistent with the idea that mifepristone increases lifespan, at least in part, through inhibition of midgut lipid metabolism and midgut hypertrophy. The *Drosophila* Dhr96 hormone receptor is related to the human Vitamin-D receptor and regulates midgut lipid uptake and metabolism [39,40,41,42]. Null mutation of *Dhr96* increased lifespan and reduced the effects of mifepristone on lifespan, suggesting a possible common mechanism [13]. Consistent with this conclusion, lipidomics analysis showed that mifepristone decreased whole-body triglyceride levels in both virgin females and mated females [13]. Mifepristone also reduced midgut lipid staining in mated females [26], and in flies of unknown sex [10]. Taken together, the data suggest a model where mifepristone acts to inhibit Eip75B function, which in turn reduces midgut lipid metabolism in both virgin females and mated females, and inhibits midgut hypertrophy in mated females. Consistent with the idea that midgut lipid metabolism limits female lifespan, previous studies showed that the lipid metabolism inhibitor etomoxir produces small lifespan increases in both virgin females and mated females but not males [30].

It is important to note that the results might also be consistent with activation of Eip75B by mifepristone. Zipper et al. have reported that RNAi inhibition of Eip75B, as well as over-expression of Eip75B, can both block midgut hypertrophy [15]. Specifically, they reported that over-expression of Eip75B drove differentiation and depletion of ISCs, thereby preventing midgut hypertrophy. For this reason, it appears possible that either inhibition or activation of Eip75B activity by mifepristone is consistent with a block of midgut hypertrophy. It is also possible that mifepristone might act on Eip75B with a mechanism similar to its interactions with the human progesterone receptor and the human glucocorticoid receptor. In the presence of progesterone, mifepristone acts as an antagonist of the human progesterone receptor, but in the absence of progesterone, mifepristone acts as a partial agonist [74,75]. Similarly, mifepristone has partial agonist activity for the human glucocorticoid receptor [76]. Conceivably, mifepristone could have either inhibitory or partial activating activity for *Drosophila* Eip75B depending upon the expression level of Eip75B, the cell type, and the presence/absence of endogenous ligands and receptor co-regulators.

The qPCR analysis confirmed a dramatic induction of GAL4 mRNA upon FLP-out activation of the *UAS-Flp-out-GAL4* transgene, as expected. However, we did not detect significant knock-down of Eip75B mRNA levels upon activation of the *UAS-Eip75B-RNAi* transgene. Consistent with this result, there is limited published data using qPCR to confirm Eip75B knockdown by RNAi. Certain studies have used qPCR to show increased Eip75B mRNA levels in response to mating [15,16]. However, those studies used loss of Eip75B functions, such as loss of ISC proliferation and/or midgut hypertrophy, to confirm Eip75B knockdown by RNAi. One study by Hoedjes et al. used the Gene-Switch system and the *da*-Gene-Switch driver to drive expression of four different *UAS-Eip75B-RNAi* constructs in mated adult females [49], including the specific VDRC#v44851 line used here. They reported a partial knockdown of whole-body Eip75B mRNA levels (less than 30% reduction) for three out of four constructs but not for VDRC#v44851. However, that study did not control for possible effects of the mifepristone on Eip75B mRNA levels. Given that mifepristone reduces egg production ([13,49]; this study), and the developing egg chambers are the main source of ecdysone upon mating [16,18,77,78], and it is ecdysone that stimulates expression of Eip75B (Ecdysone-inducible protein 75B) [16,79,80,81], it seems possible that the reductions in Eip75B mRNA observed were due to mifepristone effects as opposed to effective RNAi. Given the robust inhibition of Eip75B functions observed here and in the other studies (including loss of midgut hypertrophy), we conclude that high levels of Eip75B mRNA from one or more tissues that are not efficiently targeted by the RNAi may be obscuring a loss of Eip75B expression in some critical tissue(s) where the RNAi is more effective. For example, the GAL4/UAS system is not effective in germ-line cells, and germ-line cells are known to express Eip75B. In addition, whereas FLP-out is reported to be effective in both dividing and non-dividing cells [82], it is possible that it might be relatively more effective in some critical dividing cells, such as intestinal stem cells (ISCs).

## 5. Conclusions

Here, the conditional inhibition of *Drosophila* Eip75B was found to reduce lifespan and egg production. In addition, inhibition of Eip75B eliminated the effects of mating on midgut hypertrophy and lifespan and also eliminated the effects of mifepristone on lifespan, midgut hypertrophy, and egg production. These results have several potential implications for human biology and healthspan. The *Drosophila* Eip75B hormone receptor is related to the human hormone receptors PPARγ and REV-ERB [38]. PPARγ is known to regulate human gut metabolism, and mifepristone is reported to be a mammalian PPARγ agonist that activates expression of PPARγ target genes [11,12]. This is consistent with the hypothesis that *Drosophila* Eip75B may be a direct target of mifepristone, and this will be an important area for future studies. Gut plasticity is emerging as an important factor in human health. Obesity, late-age colon cancer, and gastrointestinal (GI) disorders are more common in women than in men, including GI disorders associated with pregnancy [83,84,85]. Lactation is associated with dramatic remodeling and growth of the intestine in female mammals [86,87], reminiscent of mating-induced midgut hypertrophy in female *Drosophila*. Recently, high dietary fructose was reported to cause increased gut size in the mouse [88], and interestingly, mifepristone prevents lipid abnormalities and insulin resistance caused by a high fructose diet in mice [89]. Anti-obesity and anti-diabetic effects of mifepristone have been observed in both in humans and mice [4,5,6,7] For example, short-term treatment with mifepristone improved adipose and hepatic insulin sensitivity in obese human patients with hyperglycemia [6]. In addition, mifepristone treatment of mice caused decreased fat mass and increased fecal lipid levels consistent with decreased lipid uptake [10]. These benefits of mifepristone in humans and mice have sometimes been attributed to mifepristone antagonism of the GR [6,10], however this has not been confirmed by GR knockdown, and it remains possible that PPARγ is a relevant mifepristone target. Possible effects of mifepristone on human gut plasticity may be an interesting area for future studies. Mifepristone use is steadily increasing in the human population. In addition to its long history of use for birth control and for treatment of Cushing’s disease, mifepristone is also being studied as a potential treatment for cancer, psychiatric conditions, alcohol abuse disorder, and other conditions [3,90,91], so the possible effects on human aging and lifespan are increasingly relevant. In the future, it may be important to further leverage the *Drosophila* model to determine the mechanisms and the potentially multiple targets of mifepristone in regulation of lifespan and healthspan.

## Figures and Tables

**Table 1:** Effect of transgene activation on lifespan in response to mating and mifepristone. Mif = 200 µg/mL mifepristone. FO-GAL4 = FLP-out-GAL4. Log-rank test comparisons are in this order: MF vs. VF; MF + Mif vs. MF; EVF vs. CVF; MF + Mif vs. VF (indicated in parentheses). The Bonferroni-corrected *p* value for significance with three comparisons is *p* < 0.0167. Significant *p* values are indicated in bold font.

Experiment	Genotype	Status	Drug	N	Med	90% Mort	Med (%)	*p*
Control 1	*Hsp70-FLP*; *75B-RNAi*	VF	-	93	48	56		
Control 1	*Hsp70-FLP*; *75B-RNAi*	MF	-	99	42	52	−12.5	**0.0012**
Control 1	*Hsp70-FLP*; *75B-RNAi*	MF	Mif	100	50	56	19.0 (4.2)	**0.0003** (0.7600)
Experimental 1	*Hsp70-FLP*; *FO-GAL4*; *75B-RNAi*	VF	-	72	45	51	−6.25	**0.0015**
Experimental 1	*Hsp70-FLP*; *FO-GAL4*; *75B-RNAi*	MF	-	66	37	54	−17.8	0.7293
Experimental 1	*Hsp70-FLP*; *FO-GAL4*; *75B-RNAi*	MF	Mif	77	41	53	10.8 (−8.9)	0.7600 (0.6208)
Control 2	*Hsp70-FLP*; *75B-RNAi*	VF	-	100	51	58		
Control 2	*Hsp70-FLP*; *75B-RNAi*	MF	-	91	40	52	−21.6	**9.02 × 10^−7^**
Control 2	*Hsp70-FLP*; *75B-RNAi*	MF	Mif	92	52	58	30.0 (1.96)	**1.51 × 10^−10^** (0.1182)
Experimental 2	*Hsp70-FLP*; *FO-GAL4*; *75B-RNAi*	VF	-	99	46	54	−9.80	**0.0030**
Experimental 2	*Hsp70-FLP*; *FO-GAL4*; *75B-RNAi*	MF	-	87	40	54	−13.0	0.6369
Experimental 2	*Hsp70-FLP*; *FO-GAL4*; *75B-RNAi*	MF	Mif	83	34	52	−15.0 (−26.0)	0.0872 (0.0234)

**Table 2:** COX-PHA of effect of transgene activation on lifespan in response to mating and mifepristone. FOG4 = FLP-out-GAL4.

**Call**: coxph(formula = (Surv(Day) ~ FOG4 + Mating + Mif + FOG4:Mating + FOG4:mif), data = new_E75BC1C2)					
*n* = 1059, number of events = 1059					
	coef	exp(coef)	se(coef)	z	Pr(>|z|)
FOG4	0.4689	1.5982	0.1065	4.404	1.06 × 10^−5^ ***
Mating	0.6941	2.0018	0.1048	6.624	3.49 × 10^−11^ ***
Mif	−0.8364	0.4333	0.1058	−7.903	2.73 × 10^−15^ ***
FOG4:Mating	−0.6476	0.5233	0.1535	−4.220	2.44 × 10^−5^ ***
FOG4:Mif	1.0368	2.8202	0.1558	6.655	2.83 × 10^−11^ ***
---					
Signif. codes: 0 ‘***’ 0.001 ‘**’ 0.01 ‘*’ 0.05 ‘.’ 0.1 ‘ ’ 1					
	exp(coef)	exp(-coef)	lower 0.95	upper 0.95	
FOG4	1.5982	0.6257	1.2972	1.9691	
Mating	2.0018	0.4995	1.6302	2.4582	
Mif	0.4333	2.3080	0.3521	0.5332	
FOG4:Mating	0.5233	1.9110	0.3874	0.7069	
FOG4:Mif	2.8202	0.3546	2.0781	3.8272	
---					
Concordance = 0.602 (se = 0.01)					
Likelihood ratio test = 113.2 on 5 df, *p* = <2 × 10^−16^					
Wald test = 109 on 5 df, *p* = <2 × 10^−16^					
Score (logrank) test = 112.9 on 5 df, *p* = <2 × 10^−16^					

**Table 3:** Effect of FLP recombination on lifespan in response to mating and mifepristone. Mif = 200 µg/mL mifepristone. FO-GAL4 = FLP-out-GAL4. Log-rank test comparisons are in this order: MF vs. VF; MF + Mif vs. MF; EVF vs. CVF EVF vs. CVF; MF + Mif vs. VF (indicated in parentheses). The Bonferroni-corrected *p* value for significance with three comparisons is *p* < 0.0125. Significant *p* values are indicated in bold font.

Experiment	Genotype	Status	Drug	N	Med	90% Mort	ΔMed (%)	*p*
Control 1	*Hsp70-FLP*	VF	-	98	47	51		
Control 1	*Hsp70-FLP*	MF	-	90	36	47	−23.4	**1.32 × 10^−7^**
Control 1	*Hsp70-FLP*	MF	Mif	89	47	56	30.1 (0.00)	**5.15 × 10^−8^** (0.1976)
Experimental 1	*Hsp70-FLP*; *FO-GAL4*	VF	-	100	44	49	−6.38	**0.0061**
Experimental 1	*Hsp70-FLP*; *FO-GAL4*	MF	-	93	32	42	−27.3	**5.81 × 10^−17^**
Experimental 1	*Hsp70-FLP*; *FO-GAL4*	MF	Mif	86	42	49	31.3 (−4.5)	**2.61 × 10^−8^** (0.3810)
Control 2	*Hsp70-FLP*	VF	-	96	47	54		
Control 2	*Hsp70-FLP*	MF	-	93	34	51	−27.7	**0.0115**
Control 2	*Hsp70-FLP*	MF	Mif	97	47	54	38.2 (0.00)	**0.0004** (0.1576)
Experimental 2	*Hsp70-FLP*; *FO-GAL4*	VF	-	96	47	54	0.00	**0.8256**
Experimental 2	*Hsp70-FLP*; *FO-GAL4*	MF	-	91	38	47	−19.1	**7.74 × 10^−13^**
Experimental 2	*Hsp70-FLP*; *FO-GAL4*	MF	Mif	82	44	49	15.8 (−6.4)	**9.88 × 10^−7^** (0.0225)

**Table 4:** COX-PHA of effect of FLP recombination on lifespan in response to mating and mifepristone. FOG4 = FLP-out-GAL4.

**Call**: coxph(formula = (Surv(Day) ~ FOG4 + Mating + Mif + FOG4:Mating + FOG4:Mif), data = new_ctrlC1C2)					
n = 1135, number of events = 1135					
	coef	exp(coef)	se(coef)	z	Pr(>|z|)
FOG4	0.17199	1.18766	0.10142	1.696	0.089921
Mating	0.57921	1.78462	0.10293	5.627	1.83 × 10^−8^ ***
Mif	−0.80469	0.44723	0.10400	−7.737	1.01 × 10^−14^ ***
FOG4:Mating	0.49064	1.63337	0.14640	3.351	0.000804 ***
FOG4:Mif	0.03748	1.03819	0.14754	0.254	0.799448
---					
Signif. codes: 0 ‘***’ 0.001 ‘**’ 0.01 ‘*’ 0.05 ‘.’ 0.1 ‘ ’ 1					
	exp(coef)	exp(-coef)	lower 0.95	upper 0.95	
FOG4	1.1877	0.8420	0.9736	1.4488	
Mating	1.7846	0.5603	1.4586	2.1835	
Mif	0.4472	2.2360	0.3648	0.5483	
FOG4:Mating	1.6334	0.6122	1.2259	2.1762	
FOG4:Mif	1.0382	0.9632	0.7775	1.3863	
---					
Concordance = 0.64 (se = 0.01)					
Likelihood ratio test = 203.5 on 5 df, *p* = <2 × 10^−16^					
Wald test = 219.2 on 5 df, *p* = <2 × 10^−16^					
Score (logrank) test = 236.9 on 5 df, *p* = <2 × 10^−16^					

## Data Availability

The raw data supporting the conclusions of this article will be made available by the authors on request.

## Supplementary Materials

The following supporting information can be downloaded at: https://www.mdpi.com/article/10.3390/cells13131123/s1, Figure S1. Conducting heat pulse and FLP-out in young adult females eliminates the effect of mating and mifepristone independent of target transgene; Table S1. Statistical summary. Table S2. Two-way ANOVA of VF vs. MF. Table S3. Two-way ANOVA of (−) Mif vs. (+) Mif. Figure S2. Maximum midgut diameter assay in control group females. Figure S3. Maximum midgut diameter assay in experimental group females. Figure S4. Quantitative real-time PCR analysis.
