# Supplementary material for: Conditional Inhibition of Eip75B Eliminates the Effects of Mating and Mifepristone on Lifespan in Female Drosophila
Source: Cells. 2024 Jun 28;13(13):1123. doi: 10.3390/cells13131123 (PMC11240670; doi:10.3390/cells13131123)
Supplement: Supplementary file 1 [file cells-13-01123-s001.zip › cells-3030794-supplementary.pdf]

Supplementary information

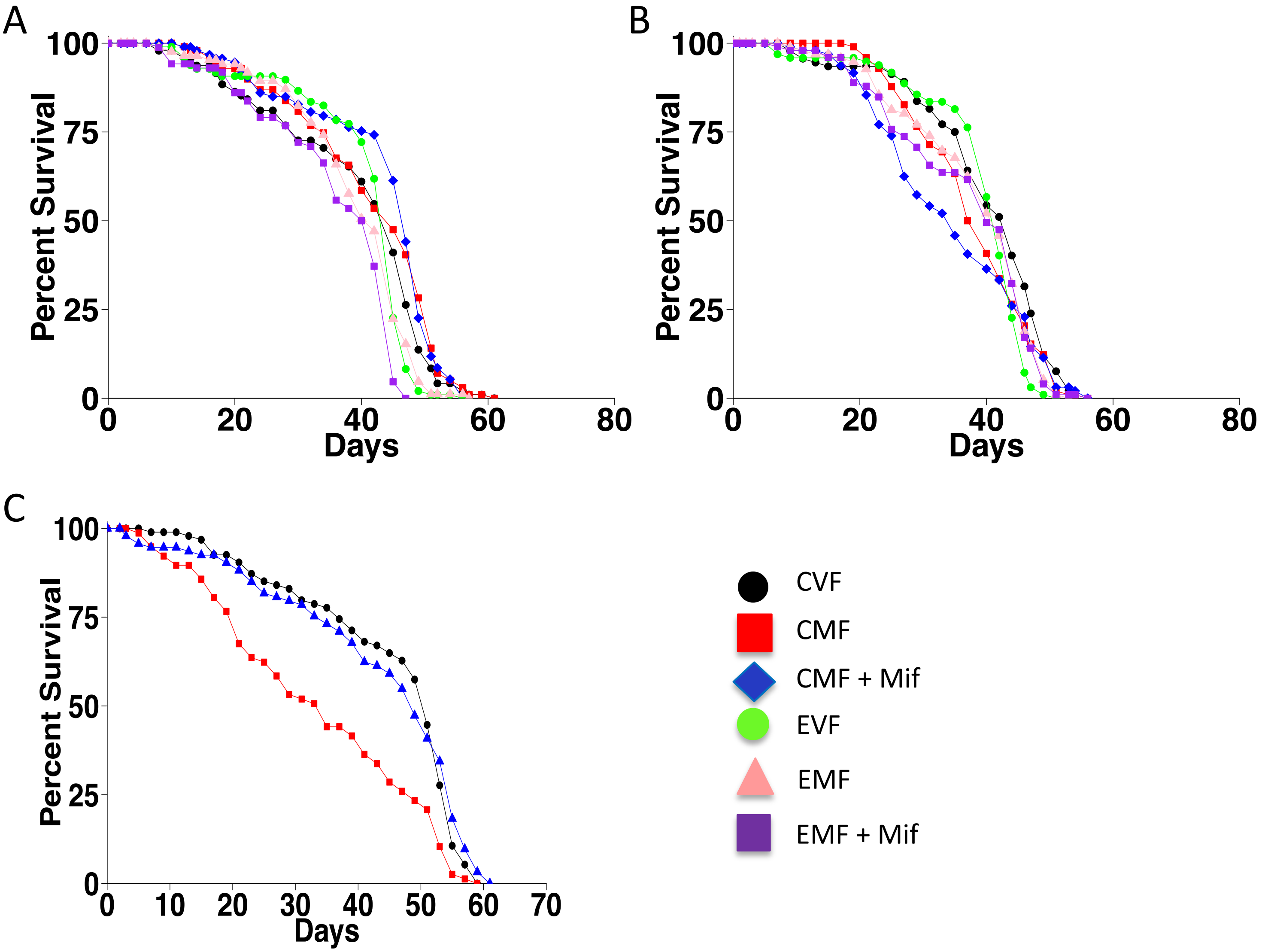

Figure S1. Conducting heat pulse and FLP-out in young adult females eliminates the effect of mating and mifepristone independent of target transgene. **(A, B**) Flies subjected to adult heat pulse. All flies were subjected to heat pulses at adult age day 2 and day 3. Virgin females (VF), mated females (MF), and mated females treated with mifepristone (MF + Mif), were assayed for life span, in two replicate experiments. Control group females (CVF and CMF) contained control chromosome. Genotype *yw HS-FLP12/ w[1118] ; UAS-75B-RNAi /+.* Experimental group females (EVF and EMF) contained isogenic chromosome with FLP-out target transgene. Genotype *yw HS-FLP12/ w[1118] FLP-out-GAL4 ; UAS-75B-RNAi /+.* (**A**) Experiment 1. (**B**) Experiment 2. (**C**) No-heat pulse controls. Control group females (CVF and CMF) contained control chromosome. Genotype *yw HS-FLP12/ w[1118] ; UAS-75B-RNAi /+.* Statistical summary presented in *Table S1*.

Table S1. Effect of FLP-out activation of UAS-Eip75B-RNAi on life span in response to mating and mifepristone in young adult flies. FO-GAL4 = Flp-out-GAL4. Log-rank test comparisons are in this order: MF vs VF; MF + Mif vs MF.

Experiment	Genotype	Status	Drug	N	Med	ΔMed (%)	*p*
Control 1	Hsp70-FLP; 75B-RNAi	VF	-	95	45		
Control 1	Hsp70-FLP; 75B-RNAi	MF	-	99	45	0.00	0.1729
Control 1	Hsp70-FLP; 75B-RNAi	MF	Mif	93	47	4.44	0.7945
Experimental 1	Hsp70-FLP; FO-GAL4; 75B-RNAi	VF	-	97	45		
Experimental 1	Hsp70-FLP; FO-GAL4; 75B-RNAi	MF	-	85	42	-6.67	0.6396
Experimental 1	Hsp70-FLP; FO-GAL4; 75B-RNAi	MF	Mif	86	41	-2.38	**0.0174**
Control 2	Hsp70-FLP; 75B-RNAi	VF	-	92	44		
Control 2	Hsp70-FLP; 75B-RNAi	MF	-	98	39	-12.5	0.1009
Control 2	Hsp70-FLP; 75B-RNAi	MF	Mif	96	35	-9.09	0.4122
Experimental 2	Hsp70-FLP; FO-GAL4; 75B-RNAi	VF	-	97	42		
Experimental 2	Hsp70-FLP; FO-GAL4; 75B-RNAi	MF	-	96	42	0.00	0.2508
Experimental 2	Hsp70-FLP; FO-GAL4; 75B-RNAi	MF	Mif	99	40	-4.76	0.8773
Control 3 (no heat pulse)	Hsp70-FLP; 75B-RNAi	VF	-	94	56		
Control 3 (no heat pulse)	Hsp70-FLP; 75B-RNAi	MF	-	77	54	-31.3	**5.17E-5**
Control 3 (no heat pulse)	Hsp70-FLP; 75B-RNAi	MF	Mif	93	57	40.0	**2.69E-5**

Table S2. 2-way ANOVA of VF vs MF

Table Analyzed	Data 1				
**Two-way ANOVA**	Ordinary				
**Alpha**	0.05				
**Source of Variation**	% of total variation	P value	P value summary	Significant?	
**Interaction**	8.449	0.0025	**	Yes	
**Row Factor (effect of mating)**	6.702	0.0068	**	Yes	
**Column Factor (effect of genotype)**	5.254	0.0161	*	Yes	
**ANOVA table**	SS (Type III)	DF	MS	F (DFn, DFd)	P value
**Interaction**	0.007985	1	0.007985	F (1, 92) = 9.660	P=0.0025
**Row Factor (effect of mating)**	0.006335	1	0.006335	F (1, 92) = 7.663	P=0.0068
**Column Factor (effect of genotype)**	0.004966	1	0.004966	F (1, 92) = 6.007	P=0.0161
**Residual**	0.07605	92	0.0008267		
**Difference between column means**					
**Predicted (LS) mean of w[1118]**	0.2033				
**Predicted (LS) mean of FOG4**	0.1887				
**Difference between predicted means**	0.01455				
**SE of difference**	0.005935				
**95% CI of difference**	0.002759 to 0.02633				
**Difference between row means**					
**Predicted (LS) mean of Virgin**	0.1878				
**Predicted (LS) mean of Mated**	0.2042				
**Difference between predicted means**	-0.01643				
**SE of difference**	0.005935				
**95% CI of difference**	-0.02822 to -0.004642				
**Interaction CI**					
**Mean diff, A1 - B1**	-0.003900				
**Mean diff, A2 - B2**	0.03299				
**(A1 -B1) - (A2 - B2)**	-0.03689				
**95% CI of difference**	-0.06047 to -0.01332				
**(B1 - A1) - (B2 - A2)**	0.03689				
**95% CI of difference**	0.01332 to 0.06047				
**Data summary**					
**Number of columns (Column Factor)**	2				
**Number of rows (Row Factor)**	2				
**Number of values**	96				

Table S3. 2-way ANOVA of (-) Mif vs (+) Mif

Table Analyzed	Data 1				
Two-way ANOVA	Ordinary				
Alpha	0.05				
Source of Variation	% of total variation	P value	P value summary	Significant?	
Interaction	3.587	0.0474	*	Yes	
Row Factor (effect of Mif)	4.175	0.0327	*	Yes	
Column Factor (effect of genotype)	10.31	0.0010	***	Yes	
ANOVA table	SS (Type III)	DF	MS	F (DFn, DFd)	P value
Interaction	0.003469	1	0.003469	F (1, 92) = 4.040	P=0.0474
Row Factor (effect of Mif)	0.004037	1	0.004037	F (1, 92) = 4.702	P=0.0327
Column Factor (effect of genotype)	0.009968	1	0.009968	F (1, 92) = 11.61	P=0.0010
Residual	0.07900	92	0.0008587		
Difference between column means					
Predicted (LS) mean of w[1118]	0.2080				
Predicted (LS) mean of FOG4	0.1873				
Difference between predicted means	0.02075				
SE of difference	0.006091				
95% CI of difference	0.008654 to 0.03285				
Difference between row means					
Predicted (LS) mean of (-) Mif	0.2042				
Predicted (LS) mean of (+) Mif	0.1910				
Difference between predicted means	0.01321				
SE of difference	0.006091				
95% CI of difference	0.001110 to 0.02530				
Interaction CI					
Mean diff, A1 - B1	0.03299				
Mean diff, A2 - B2	0.008509				
(A1 -B1) - (A2 - B2)	0.02449				
95% CI of difference	0.0002917 to 0.04868				
(B1 - A1) - (B2 - A2)	-0.02449				
95% CI of difference	-0.04868 to -0.0002917				
Data summary					
Number of columns (Column Factor)	2				
Number of rows (Row Factor)	2				
Number of values	96				

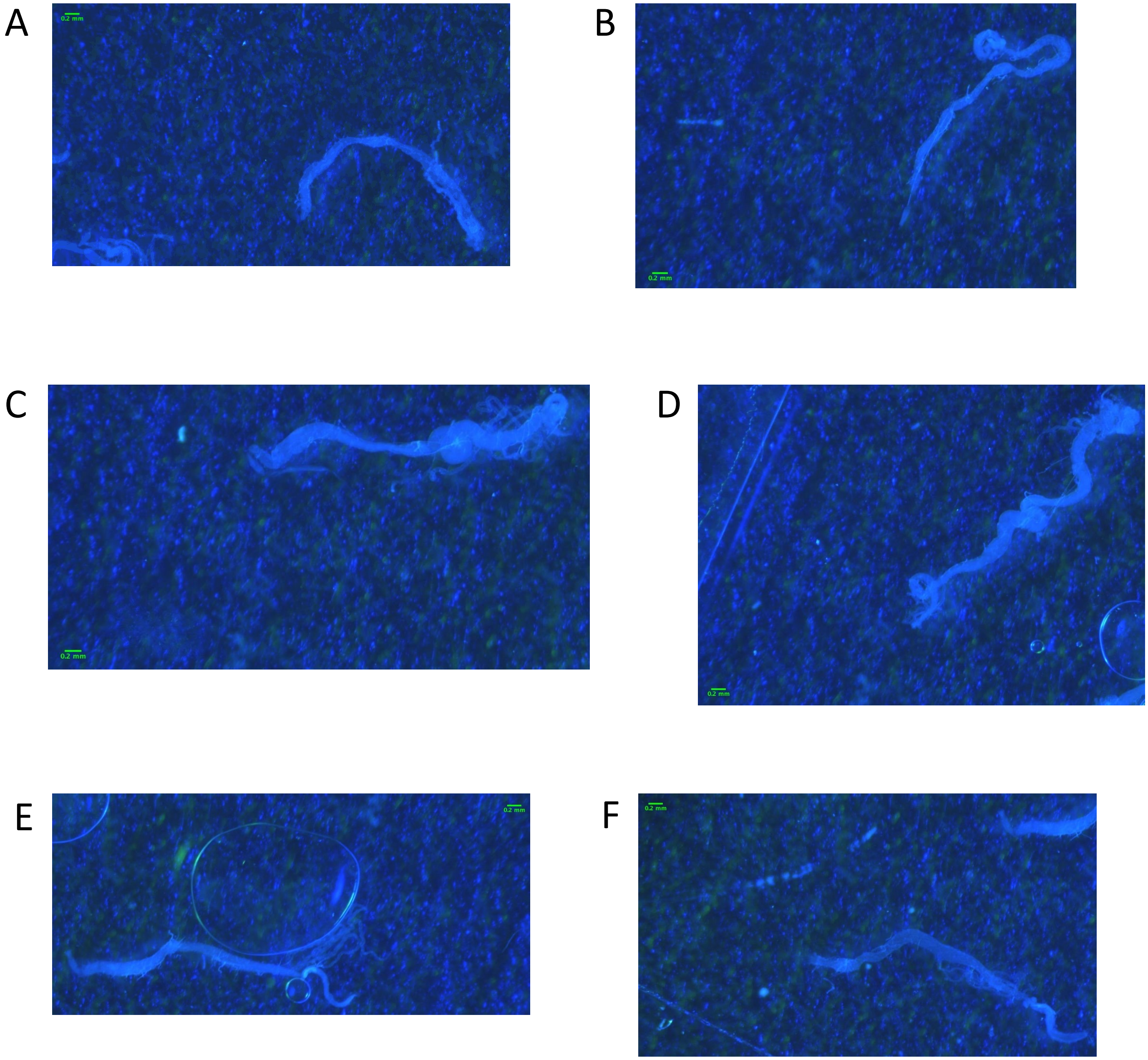

Figure S2. Maximum midgut diameter assay in control group females. Representative images of dissected midgut tissue from the experiment of Figure 1. All flies were subjected to heat pulses at third larval instar stage. Virgin females (VF), mated females (MF), and mated females treated with mifepristone (MF + Mif) were then assayed for maximum midgut diameter at age 14 days. Control group females (CVF, CMF) contain the control chromosome. Genotype yw HS-FLP12/ w[1118] ; UAS-75B-RNAi /+. (**A, B**) CVF. (**C, D**) CMF. (**E, F**) CMF + Mif.

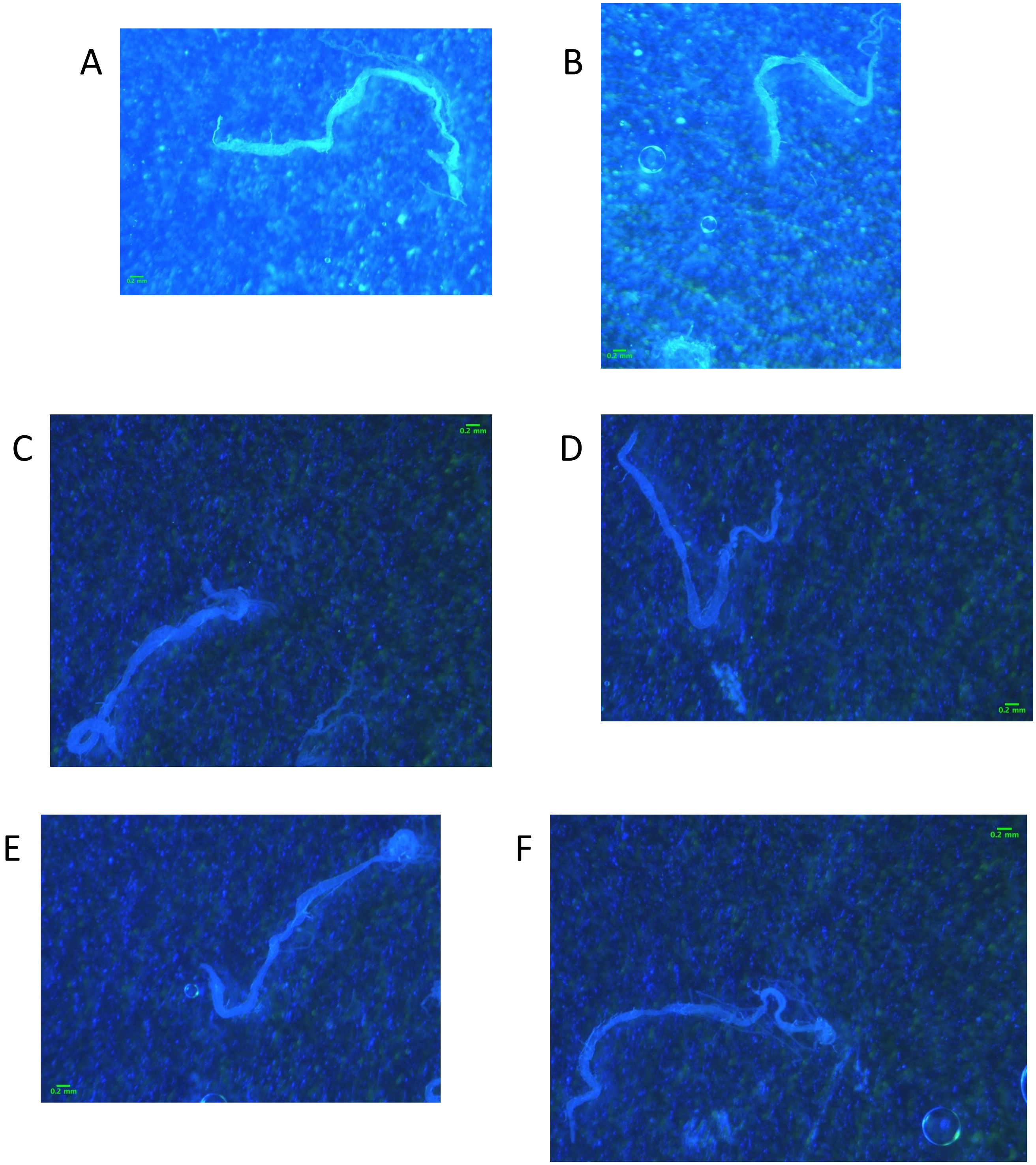

Figure S3. Maximum midgut diameter assay in experimental group females. Representative images of dissected midgut tissue from the experiment of Figure 1. All flies were subjected to heat pulses at third larval instar stage. Virgin females (VF), mated females (MF), and mated females treated with mifepristone (MF + Mif) were then assayed for maximum midgut diameter at age 14 days. Experimental group females (EVF, EMF) contain the isogenic chromosome with FLP-out target transgene. Genotype yw HS-FLP12/ w[1118] FLP-out-GAL4 ; UAS-75B-RNAi /+. (**A, B**) EVF. (**B, C**) EMF. (**D, E**) EMF + Mif.

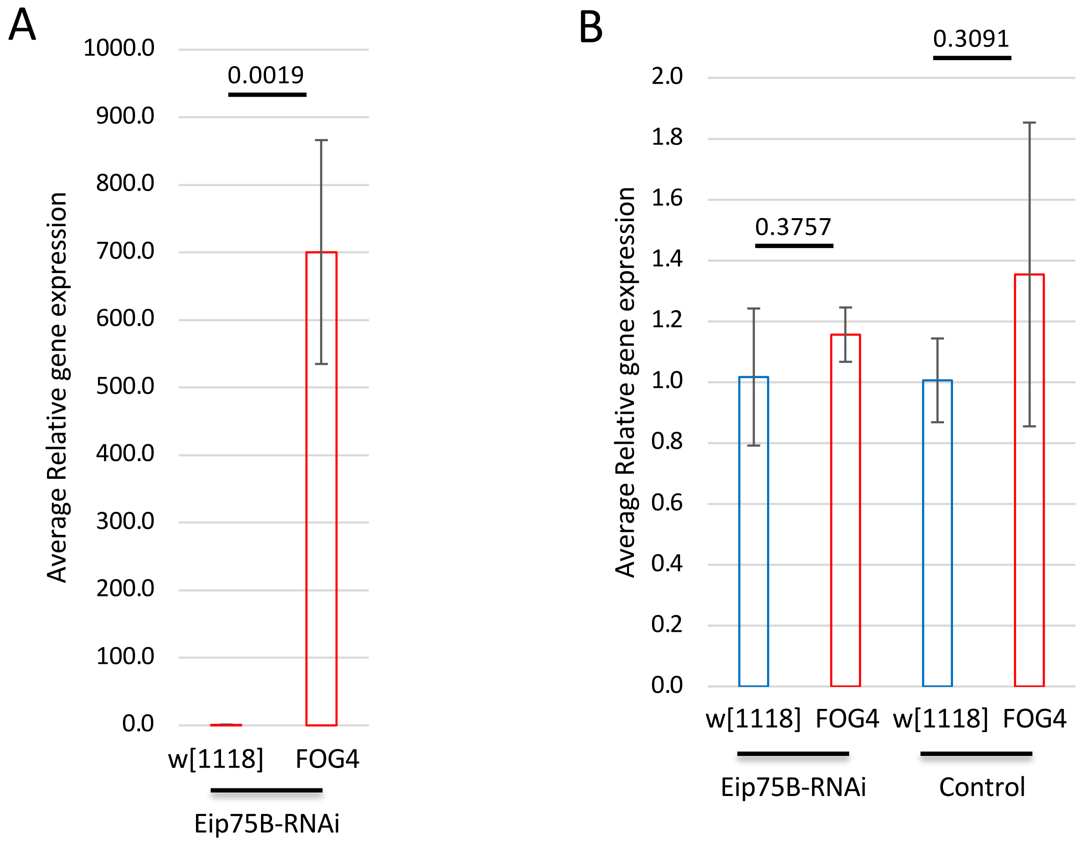

Figure S4. Quantitative real-time PCR analysis. Gene expression was analyzed in whole-body RNA isolated from mated females at 14 days of age. Transcript levels were normalized to TBP. For the "Eip75B-RNAi" containing genotypes, the "w[1118]" group contained the control chromosome, genotype *yw HS-FLP12/ w[1118] ; UAS-75B-RNAi /+,* and the "FOG4" group contained the isogenic FLP-out target transgene, genotype *yw HS-FLP12/ w[1118] FLP-out-GAL4 ; UAS-75B-RNAi /+.* For the "Control" genotypes, the *UAS-Eip75B-RNAi* transgene was not present. The *"w[1118]"* group contained the control chromosome, genotype *yw HS-FLP12/ w[1118] ; Sco /+,* and the *"FOG4"* group contained the isogenic chromosome with FLP-out target transgene, genotype *yw HS-FLP12/ w[1118] FLP-out-GAL4 ; Sco /+.* (**A**) UAS-GAL4 transgene expression. GAL4 expression quantified using GAL4-specific primers. (**B**) Eip75B endogenous gene expression. Each bar represents the average +/- Standard Deviation of 3 technical replicates for each of 6 biological replicates, for a total of 18 values. The statistical test is unpaired two-sided t-test, and the Bonferroni-corrected *p* value for significance with one comparisons is $p < 0.05$.
